# Comparative Genomics of Herpesviridae Family to Look for Potential Signatures of Human Infecting Strains 

**DOI:** 10.1155/2016/9543274

**Published:** 2016-05-26

**Authors:** Vikas Sharma, Fauzul Mobeen, Tulika Prakash

**Affiliations:** School of Basic Sciences, Indian Institute of Technology, Mandi 175005, India

## Abstract

Herpesviridae family is one of the significant viral families which comprises major pathogens of a wide range of hosts. This family includes at least eight species of viruses which are known to infect humans. This family has evolved 180–220 million years ago and the present study highlights that it is still evolving and more genes can be added to the repertoire of this family. In addition, its core-genome includes important viral proteins including glycoprotein B and helicase. Most of the infections caused by human herpesviruses have no definitive cure; thus, search for new therapeutic strategies is necessary. The present study finds core-genome of human herpesviruses that differs from that of Herpesviridae family and nonhuman herpes strains of this family and might be a putative target for vaccine development. The phylogenetic reconstruction based upon the protein sequences of core gene set of Herpesviridae family reveals the sharp splits of its different subfamilies and supports the hypothesis of coevolution of viruses with their hosts. In addition, data mining for cis-elements in the genomes of human herpesviruses results in the prediction of numerous regulatory elements which can be used for regulating the expression of viral based vectors implicated in gene therapies.

## 1. Introduction

Human herpesviruses (HHVs) are one of the major human pathogens and are known to cause several diseases including herpes genitalis, infectious mononucleosis, and Kaposi's sarcoma. Herpes simplex virus type 1 (HSV-1) and herpes simplex virus type 2 (HSV-2) are the most common pathogens among HHVs and cause several infections including genital or oral herpes, conjunctivitis, and encephalitis, commonly known as herpes simplex infection. This infection is incurable and around 90% of world's population is infected with one or both viruses [1]. If human simplex virus (HSV) induced encephalitis remains untreated, it has a very high (>70%) fatality rate [2]. Its management is also poor which results in death of a major proportion of patients while only a minor proportion returns to normal functions. In addition, Epstein-Barr virus (EBV) is another one of most common human pathogens and is implicated in a number of human malignancies. Previous study showed that EBV-attributable malignancies accounted for 1.8% of all cancer deaths in 2010 and this percentage is increased by 14.6% over a period of 20 years [3]. There are no definitive therapies or drugs available for most of the HHV induced infections. Global burden of HHV induced infections is increasing rapidly which needs effective means of prognosis and therapeutics for its better management. On the other side, few members of HHVs including HSVs are also implicated as vectors for vaccine development and gene therapy of several diseases, namely, Parkinson's disease and Alzheimer's disease. Cis-elements play significant role in the regulation of these virus-vectors for desired gene expression. These aspects of HHVs make them significant for clinical and pharmaceutical research.

HHVs belong to Herpesviridae family of Herpesvirales order under group I (dsDNA) in virus classification hierarchy. Members of Herpesviridae family are well characterized and are known to infect a wide range of hosts. In addition to humans, these hosts include mammals, birds, reptiles, amphibians, molluscs, and fish. At least eight species of HHVs are found to infect humans. Based upon biological features and genomic attributes, members of Herpesviridae family have been classified into three subfamilies including Alphaherpesvirinae, Betaherpesvirinae, and Gammaherpesvirinae with their estimated origin being 180 to 220 million years ago [4]. The Alphaherpesvirinae subfamily includes important HHVs, namely, HSV-1, HSV-2 and* Varicella zoster* virus (*V. zoster* virus). The Betaherpesvirinae subfamily includes human* Cytomegalovirus* (HCMV), human herpesvirus 6 (HHV-6A and HHV-6B), and human herpesvirus 7 (HHV-7) whereas the Gammaherpesvirinae subfamily includes the rest of HHVs, namely, Epstein-Barr virus (HHV-4) and human herpesvirus 8 (HHV-8). For detailed insights into the taxonomic and genomic attributes of the herpesvirus family the readers are advised to refer to the comprehensive study by Davison [5].

In recent times, a bloom in sequencing technologies has contributed to an increase in the number of publically available genome sequences of several members of Herpesviridae family. This has led us to investigate this family in context of its genomic diversity and evolutionary aspects. In this study, we performed a pan-genome analysis and phylogenetic clustering of publically available complete genomes of 64 members of Herpesviridae family. Further, a detailed analysis was conducted to explore the differentiating genomic attributes of HHVs in comparison to non-HHVs belonging to Herpesviridae family. The core gene sets of HHVs are further screened for putative antigenic determinants which might be potential candidates for epitope-based vaccine development. In addition, we also carried out genome data mining of HHVs for regulatory cis-elements which might be crucial factors for modulating the expression of viral genes in vaccine development and gene therapy for fatal diseases.

## 2. Materials and Methods

### 2.1. Materials

Publically available complete proteomes of 64 members of Herpesviridae family including 10 HHVs and 54 non-HHVs are retrieved from NCBI database (Table 1 and Supplementary Table  1 in Supplementary Material available online at http://dx.doi.org/10.1155/2016/9543274). In addition, complete genomes of 10 HHVs are also downloaded from NCBI database (Table 1).

### 2.2. Methods

#### 2.2.1. Pan-Genome Analysis

The pan-genome is calculated by using ortho Markov cluster (OMCL) and clusters of orthologous groups (COG) methods implemented in get_homologues package [7] with default parameters. The intersection of these two algorithms is taken for determination of pan-genome which includes four compartments including core (genes contained in all considered genomes), soft core (genes present in 95% of the considered genomes), cloud (genes present in a few genomes), and shell (remaining genes contained in several genomes). The expansion of a pan-genome size is examined by plotting the number of genomes considered against the total number of genes. The pan-genome plot is fitted with Tettelin function available in get_homologues package [7] to estimate the size of pan-genome. Similarly, the contraction of core-genome size is examined by plotting the number of genomes considered against the total number of genes. The core-genome plot is fitted with Tettelin function available in get_homologues package [7] to estimate the size of core-genome.

#### 2.2.2. Core-Genome Analysis

The core-genome is evaluated by using bidirectional best-hit (BDBH), OMCL, and COG clustering strategies implemented in get_homologues package [7] with default parameters. The intersection of these three clustering methods is taken as stringent consensus core-genome.

#### 2.2.3. Epitope Prediction

Data mining is done for the identification of antigenic determinants in the core gene set of HHVs using Immune Epitope Database (IEDB, http://www.iedb.org/). The core gene products of HHVs are screened for any kind of epitopes involved in any human disease which can induce human immune response. To achieve this, we have searched the IEDB database (version as on 15th April, 2016) with antigen (parameter organism: human herpesvirus species, namely, HHV-1, HHV-2, HHV-3, HHV-4, HHV-5, HHV-6 (type A and type B), HHV-7, and HHV-8) and host (humans) and using the other parameters as the default values.

#### 2.2.4. Phylogenetic Reconstruction

The phylogenetic reconstruction is done based upon core gene set of 64 members of Herpesviridae family. To achieve this, glycoprotein B (gB) and helicase protein sequences are extracted from the proteomes and were concatenated. Sequence alignment is done using ClustalW module of MEGA6 [8] with default parameters. The evolutionary history is inferred using the Neighbor-Joining method. The bootstrap consensus tree inferred from 1,000 replicates is taken to represent the evolutionary history of the taxa analysed. The evolutionary distances are computed using the JTT matrix-based method and are in the units of the number of amino acid substitutions per site. The analysis involved 64 amino acid sequences. All positions containing gaps and missing data are eliminated. There are a total of 1,283 positions in the final dataset. Evolutionary analyses are conducted in MEGA6 [8].

#### 2.2.5. Cis-Element Prediction

To predict the cis-regulatory regions in DNA sequences of HHVs, standalone version of Cister (Cis-element Cluster Finder) tool [9] is used. Default parameters of Cister tool are used along with default nucleotide count matrices for the selection of 16 cis-elements available on the given webpage (http://zlab.bu.edu/~mfrith/NucFreqMat.html).

## 3. Results and Discussion

### 3.1. Pan-Genome Analysis of Herpesviridae Family

**Figure 1:**
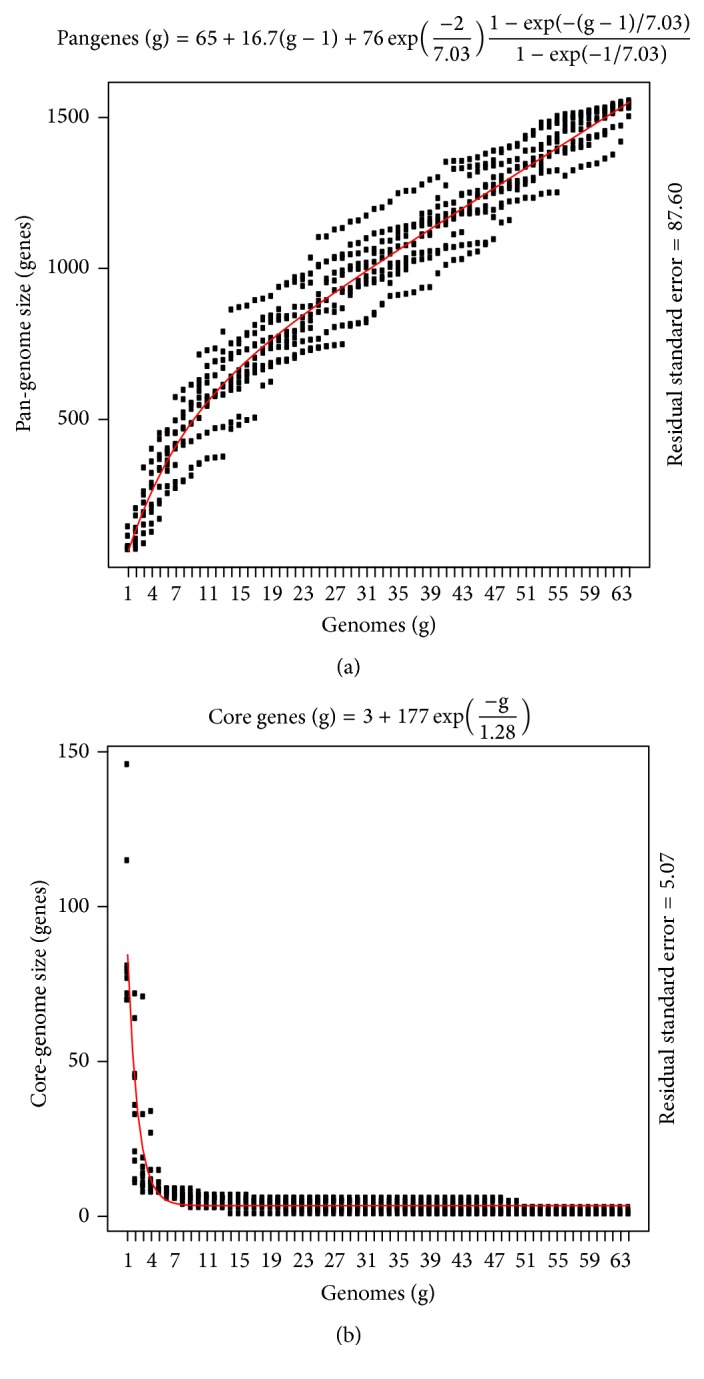
Plot of the estimation of pan- and core-genome sizes of Herpesviridae family fitted with Tettelin function. (a) Pan-genome and (b) Core-genome estimates are shown after using ten random samples of the 64 taxa. Residual standard errors are reported on the right margin as a measure of the goodness of fit.

To determine the global gene repertoire of the Herpesviridae family, the number of new genes added by each genomic sequence is estimated. The expansion of a pan-genome is examined by plotting the number of genomes considered against the pan-genes observed along with Tettelin fit. The resulting pan-genome curve suggests its open nature as it does not reach a plateau and grows by an average of 24 genes per genome (Figure 1(a)). This open pan-genome indicates the continuous evolution of Herpesviridae family using different gene acquisition strategies, namely, horizontal gene transfer and diversification. This indicates towards the expansion of the pan-genome of Herpesviridae family with the increase in the number of additionally sequenced species. The open nature of the pan-genome of Herpesviridae family is also consistent with the hypothesis that species inhabiting a wide range of environments tend to have an open pan-genome [10, 11].

Further, pan-genomes obtained by OMCL and COG algorithms produce clusters of 2,094 and 2,271 genes, respectively, whereas their intersection results in a cluster of 1,785 genes. In addition, this gene cluster is further classified into four compartments including core (0.28%), soft core (0.50%), cloud (86.94%), and shell (12.54%) (Supplementary Figure 1). The core gene set of the Herpesviridae family includes the genes present in all 64 genomes which are highly conserved [12] during the evolution of this family, whereas soft core includes the genes which are present in at least 60 genomes taken in this study. Basically, soft core estimates a more robust core with the possibility of missing or truncated genes [13]. Shell component of Herpesviridae family estimates the genes present in >2 genomes but <60 genomes and represents limited conservation [12] during the evolution of this family. The gain and loss of these genes from a given genome is supposed to occur at slower rate [14]. In contrast, cloud component includes the genes which are gained and lost from the genomes at faster rate [14] and are poorly conserved [12]. In our dataset, cloud component estimates the genes present in ≤2 viral genomes of the Herpesviridae family.

In contrast to the expansion of size of pan-genome, the contraction of core-genome size is evaluated by plotting the number of genomes considered against the core genes observed followed by fitting the plot with Tettelin function. The core-genome size of Herpesviridae family represents a well fitted decaying exponential trend (Figure 1(b)). It implies that the number of core genes present in all considered genomes tends to decrease with the addition of genomes and reaches a saturation level after finding minimal essential core set required for viral survival and growth.

### 3.2. Evaluation of Core-Genome of Herpesviridae Family

**Figure 2:**
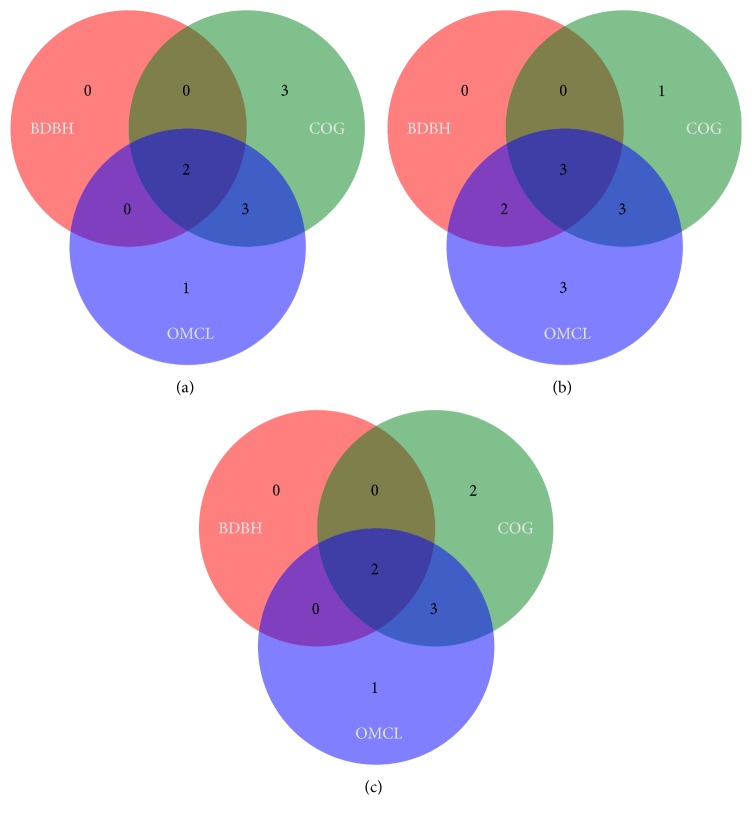
Venn diagrams of core-genomes generated by the BDBH, COG, and OMCL strategies for (a) 64 members of Herpesviridae family, (b) 10 HHVs of Herpesviridae family, and (c) 54 Non-HHVs of Herpesviridae family.

A highly stringent strategy is further employed to find a minimal essential core of Herpesviridae family. Towards this, the core-genomes are examined by three clustering methods including BDBH, COG, and OMCL strategies resulting in clusters of 2, 8, and 6 genes, respectively. whereas their intersection produces a cluster of 2 genes (Figure 2(a)). This might be a minimal set of critical genes essential for the survival of all members of Herpesviridae family taken in this study. These genes code for glycoprotein B and helicase proteins. Glycoprotein B is a primary and crucial component of the herpesvirus fusion machinery which is involved in the cell entry of herpesviruses into the host cells [15]. Similarly, helicase is another crucial component of viral genomes which is essential for several significant biological processes including viral genome replication, transcription, and translation [16].

### 3.3. Difference in the Genomic Attributes of HHVs and Non-HHVs Belonging to Herpesviridae Family

To obtain better insights into the differences in the genomic attributes of HHVs and non-HHVs of Herpesviridae family, their corresponding core-genomes are evaluated. Core-genome evaluation of HHVs is done using three clustering methods including BDBH, COG, and OMCL resulting in three different clusters consisting of 5, 7, and 11 genes, respectively (Figure 2(b)). Intersection of these three methods results in a final cluster of 3 genes which represents a minimal set of core genes of HHVs. This includes genes encoding glycoprotein B, helicase, and major capsid proteins. In case of non-HHV strains of Herpesviridae family, core-genome analysis results in BDBH, COG, and OMCL clusters of 2, 7, and 6 genes, respectively, whereas their intersection results in a cluster of 2 genes (Figure 2(c)). This minimal set of core genes of non-HHVs of Herpesviridae family encodes glycoprotein B and helicase proteins. Though the core gene set of non-HHVs of Herpesviridae family is identical to that of the whole Herpesviridae family but is different from the core gene set of HHVs, the core gene set of HHVs include one additional gene encoding major capsid protein which is absent in the core gene set of non-HHVs of Herpesviridae family. It implies that major capsid protein may be critical for the HHVs infecting humans.

Major capsid protein functions in the assembly of the capsid and DNA packaging into the capsid for new viral particles within the host [17]. A study carried out on human papillomavirus 16 [18] concluded that yield of virus-like particles (VLPs) is different in different viral strains with distinct sequences of major capsid protein L1. In addition, major capsid protein is also implicated in immunogenicity and thus becomes a suitable target for vaccine development. For instance, major capsid protein (VP1) of Merkel cell polyomavirus [19] was found to be a major immune activating factor inducing a robust polyclonal antibody response.

### 3.4. Epitope Prediction in Core Gene Set of HHVs

We have investigated putative antigenic determinants in the core gene set of HHVs which might have significant role in inducing human immune system. From Table 2, it is evident that envelope glycoprotein B of all HHVs except for human herpesvirus 7 consists of numerous epitopes, whereas antigenic determinants of the major capsid protein were identified in the genomes of all HHVs except for HHV-3, HHV-7, and HHV-8. However, no antigenic determinant is located in the helicase protein of any of the HHVs. In addition, from our analysis we have identified only three epitopes in the genome of HHV-7 which are present on the antigenic proteins including ribonucleoside-diphosphate reductase large subunit-like protein U28, other human herpesvirus 7 proteins, and protein U3. The presence of a comparatively lower number of epitopes in the genome of HHV-7 and in particular the absence of antigenic determinants in the core proteins including glycoprotein B and major capsid protein of HHV-7 indicates the limiting nature of pathogenesis of this virus (Table 2). This is corroborated by the fact that HHV-7 is not a known causative agent of any definitive disease although it has been found to be implicated in febrile seizures with or without roseola infantum infection along with HHV-6 virus [20]. In addition, reviews by Long et al., [21] and Griffiths [22] also suggest the lower pathogenicity of HHV-7.

Recently, the epitope-based vaccination has been suggested as a promising measure to enhance the protective immunity against several infections caused by HHVs and other viral species. For example, YNND epitope present in the glycoprotein B of HCMV was found to be a significant target for vaccine development in order to induce protective immunity [23]. Similarly, protective epitope peptide from glycoprotein D (gD) of HSV-1 was found to have immunomodulatory protective effects [24]. In another study, multiepitope assembly peptide (MEAP) from HSV-2 was found to induce efficient protective immune response in mice [25]. Similarly, epitope vaccine based on EBV-specific CD8+ T-cell peptide was found to be a potent vaccine against infectious mononucleosis in phase I trial [26]. Thus, the putative antigenic determinants of glycoprotein B and major capsid protein might be potential targets for epitope-based vaccine development for protective immune response against infections caused by HHVs. Although no antigenic determinant is located in helicase enzyme of HHVs, the complex of this enzyme with primase is suggested to be an efficient drug target against the infections caused by HSV-1 [27], HSV-2 [28], and HHV-3 [29]. Thus, helicase and putative antigenic determinants of glycoprotein B and major capsid protein of HHVs may become effective targets for drug and vaccine development; experimental investigations are required to develop therapeutics and drugs using these targets.

### 3.5. Phylogenetic Reconstruction

**Figure 3:**
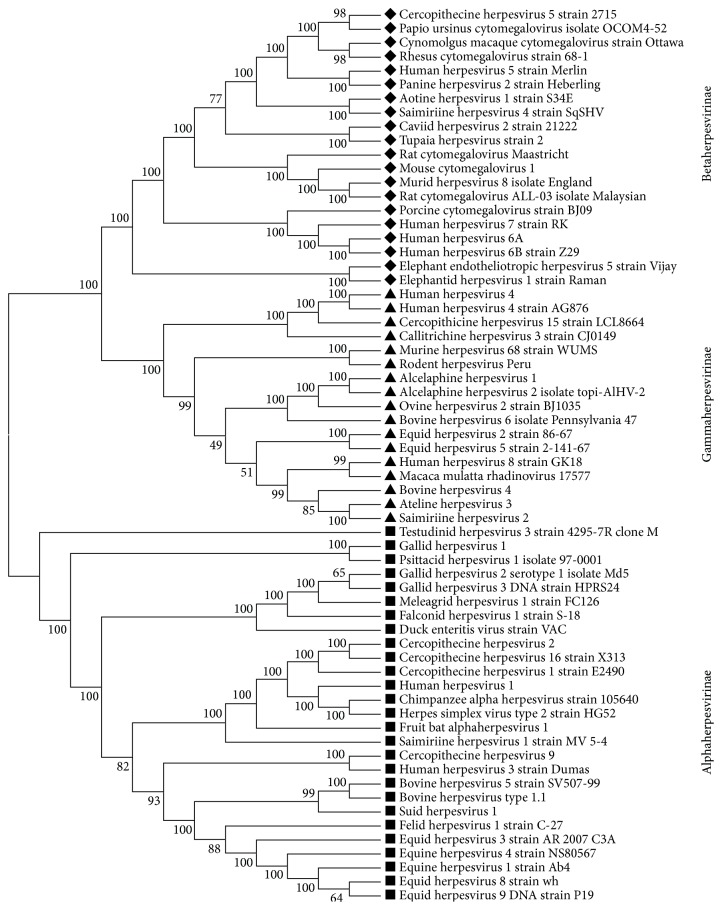
Phylogenetic tree based on the core gene set of Herpesviridae family. The percentage of replicate trees in which the associated taxa clustered together in the bootstrap test (1000 replicates) are shown next to the branches.

Phylogenetic reconstruction of Herpesviridae family is done using its core gene products including glycoprotein B and helicase proteins. The phylogenetic tree based upon these two conserved proteins clearly resolves the splits between herpesvirus subfamilies and sublineages (Figure 3). Present study supports the previous observations of early split of Betaherpesvirinae and Gammaherpesvirinae subfamilies from Alphaherpesvirinae subfamily [30]. It is also seen that a few lineages of herpesviruses are clustered together, namely,* Elephant endotheliotropic herpesvirus* 5, which is clustered with* Elephantid herpesvirus *1 and* Alcelaphine herpesvirus *1, which is clustered with* Alcelaphine herpesvirus *2. These observations are consistent with the hypothesis of coevolution of viruses with their hosts [30].

### 3.6. Data Mining of the Genomes of HHVs for the Prediction of Cis-Elements

Cis-elements are regulatory sequences, namely, promoters and enhancers, which regulate gene expression and control cellular dynamics in terms of its structures and functions. Cis-elements are usually composed of noncoding DNA and contain protein binding sites for transcription factors (TFs) or transcription regulators (TRs) which are essential to initiate and regulate the process of transcription. Cis-elements play significant role in virus induced pathogenesis by determining the range of cell-types susceptible to viral infection, modulating and resisting the host immune system [31]. In the present study, we have mined the genomes of 10 HHVs for the prediction of 16 candidate cis-elements taken in this study (Table 3). The genome of HSV-2 is found to have highest number of putative cis-elements (535), whereas the genome of human herpesvirus 7 strain RK is found to have lowest number of putative cis-elements (105). This implies that herpes simplex virus type 2 has a complex regulation system as compared to human herpesvirus 7 strain RK. This is consistent with the previous findings which show that genome of herpes simplex virus type 2 is comparatively more complex [32]. All 16 cis-elements are found to be present in the genomes of 7 HHVs. However, the genomes of 3 HHVs including human herpesvirus 3 strain Dumas, human herpesvirus 6A, and human herpesvirus 7 strain RK are found to lack 3 (AP-11, ERE, and Myf), 4 (ERE, LSF, SRF, and Tef), and 3 (AP-11, CRE, and Myc) cis-elements, respectively (Table 3).

**Figure 4:**
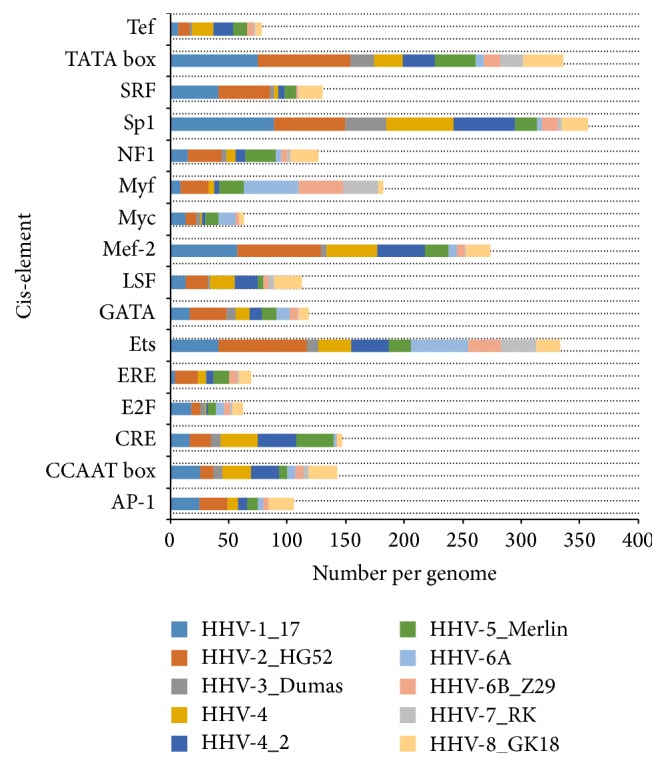
Bar-plots of count of different cis-elements in the genomes of HHVs. HHV-1_17: human herpesvirus 1; HHV-2_HG52: herpes simplex virus type 2 strain HG52; HHV-3_Dumas: human herpesvirus 3 strain Dumas; HHV-4: human herpesvirus 4; HHV-4_2: human herpesvirus 4 strain AG876; HHV-5_Merlin: human herpesvirus 5 strain Merlin; HHV-6A: human herpesvirus 6A; HHV-6B_Z29: human herpesvirus 6B strain Z29; HHV-7_RK: human herpesvirus 7 strain RK; HHV-8_GK18: human herpesvirus 8 strain GK18.

Further analysis of cis-elements shows that Sp1 is the most abundant cis-element followed by TATA, Ets, and Mef-2 cis-elements which are present in all genomes of HHVs (Figure 4 and Table 3). Sp1 is implicated in the regulation of E6 promoter activity and governs the transcriptional activity of human papillomaviruses (HPVs) in epithelial cells [31]. In addition, Sp1 is found to be an essential component of immediate early enhancers of HSVs which are implicated in upregulating the process of DNA replication [33]. TATA box is another significant cis-element and is known to be implicated in the optimal expression of glycoprotein C (gC) and late gene expression in case of HHVs. In addition, double mutation in TATA box is found to reduce viral replication and thus suggests its significance for maximal activity in adenoviruses [34]. Similarly, Ets cis-element plays significant role in the activation of early viral gene expression of human* Cytomegalovirus *(HCMV) [35]. This HCMV infection activates the pathway of mitogen-activated protein kinases/extracellular signal-regulated kinases (MAPK/ERK) which in turn regulates host cell cycle and viral pathogenesis [35]. Also, Ets cis-element is found to be involved in the latency and reactivation of herpes simplex virus 1 by stimulating its ICP0 promoter [36]. Besides these cis-elements, Mef-2 is also considered as a significant regulatory entity which recruits class II histone deacetylase upon its binding with transcription factor [37]. This enzyme determines the fate of latency in Epstein-Barr virus (EBV) and thus suggests its significance in viral life cycle [37]. In addition, this also suggests the importance of inhibitors of Mef-2 transcription factors for the reactivation of EBV [37].

## 4. Conclusions

Herpesviridae family consists of significant human pathogenic strains causing several incurable diseases. In this paper, genome based approaches have been employed to mine the putative targets with therapeutic values in the genomes of HHVs. Towards the evolutionary aspects of Herpesviridae family, pan-genome analysis shows its open nature, that is, this family is still evolving and more genes are yet to be added to its repertoire with the addition of new sequences. We have also estimated the core-genome of Herpesviridae family that differs from the core-genome of HHVs which has one additional gene encoding major capsid protein. Two genes (glycoprotein B and major capsid protein) of this core set may be used for epitope-based vaccine development, whereas third-gene encoding helicases too have target-based therapeutic values. Further, phylogenetic reconstruction based upon protein sequences of the core gene set of Herpesviridae family shows consistent results with previous studies and represents sharp splits among Alphaherpesvirinae, Betaherpesvirinae, and Gammaherpesvirinae subfamilies. In addition, cis-elements are also predicted in the genomes of HHVs which can be used as modulators of gene expression in viral-vector based gene therapies. This study is significant in context of the data mining of putative factors of HHVs which might have significant therapeutic values, although experimental investigations are required for the validation of the role of these putative factors in therapies for different diseases caused by HHVs. Being a significant viral family consisting of major human pathogens and having potential to infect a wide range of hosts, Herpesviridae family requires further research for deep insights into their evolution and pharmaceutical aspects.

## Supplementary Material

Supplementary Figure 1: Partition of the *Herpesviridae* family pan-genomic matrix into 2 shell, cloud, soft-core, and core compartments. Total gene clusters = 1,785; taxa = 64.
Supplementary Table 1: Calculation of cloud, shell, soft core and core genomes.

## Figures and Tables

**Table 1:** List of human herpesviruses and their related diseases as discussed in a review by Gilden et al. [6].

Virus species	Disease	Viral strain used in this study [NCBI accession number]	[Proteins], [nucleotide length (bp)], [GC (%)],
Alphaherpesvirinae subfamily			
HHV-1/human herpesvirus 1/herpes simplex virus 1/HSV-1	Encephalitis	Human herpesvirus 1 [NC_001806]	[77], [152,222], [68.3]
HHV-2/human herpesvirus 2/herpes simplex virus 2/HSV-2	Genital herpes, aseptic meningitis, and recurrent radiculopathy	Herpes simplex virus type 2 (strain HG52) [NC_001798]	[77], [154,675], [70.4]
HHV-3/human herpesvirus 3/*Varicella zoster* virus/human herpesvirus 3/VZV	Chickenpox (*Varicella*)	Human herpesvirus 3 (strain Dumas) [NC_001348]	[73], [124,884], [46]

Betaherpesvirinae subfamily			
HHV-5/human herpesvirus/*Cytomegalovirus*	Congenital infection, microcephaly, seizures, hypotonia, spasticity, Guillain-Barre syndrome, and acute brachial plexopathy	Human herpesvirus 5 strain Merlin [NC_006273]	[169], [235,646], [57.5]
HHV-6/human herpesvirus 6/T-cell lymphotropic virus	Roseola, febrile seizures, and fulminant hepatitis	Human herpesvirus 6A [NC_001664]	[88], [159,322], [42.4]
		Human herpesvirus 6B strain Z29 [NC_000898]	[104], [162,114], [42.8]
HHV-7/human herpesvirus 7	Encephalitis	Human herpesvirus 7 strain RK [NC_001716]	[86], [153,080], [36.2]

Gammaherpesvirinae subfamily			
HHV-4/human herpesvirus 4/Epstein-Barr virus	Aseptic meningitis, encephalomyeloneuritis, and neuritis	Human herpesvirus 4 [NC_007605]	[95], [171,823], [59.5]
		Human herpesvirus 4 strain AG876 [NC_009334]	[80], [172,764], [59.5]
HHV-8/human herpesvirus 8/Kaposi's Sarcoma-associated herpesvirus	Kaposi's sarcomas	Human herpesvirus 8 strain GK18 [NC_009333]	[86], [137,969], [53.8]

**Table 2:** List of count of putative epitopes predicted in the envelope glycoprotein B and major capsid protein in the genomes of HHVs. In addition, total number of antigenic proteins and epitopes found in the genomes of HHVs are also given.

HHV species	Number of epitopes in		Total number of	
	Envelope glycoprotein B	Major capsid protein	Antigens	Epitopes
HHV-1	20	1	29	175
HHV-2	12	7	24	253
HHV-3	5	0	17	168
HHV-4	10	3	47	765
HHV-5	45	11	85	942
HHV-6 (type A and type B)	1	9	21	63
HHV-7	0	0	3	3
HHV-8	215	0	14	433

**Table 3:** List of count of putative regulatory cis-elements predicted in the genomes of HHVs; for abbreviations of HHVs, refer to Table 1. HHV-1: HHV-1_17, HHV-2: HHV-2_HG52, HHV-3: HHV-3_Dumas, HHV-4: HHV-4, HHV-4_2: HHV-4_AG876, HHV-5: HHV-5_Merlin, HHV-6A: HHV-6A, HHV-6B: HHV-6B_Z29, HHV-7: HHV-7_RK, and HHV-8: HHV-8_GK18.

CE/TFBS	TF/DBRP or DBRD	Number in HHV-										
		1	2	3	4	4_2	5	6A	6B	7	8	Total
AP-1	TF in Fos and Jun subfam.	25	24	0	9	8	9	5	4	0	22	106
CCAAT box	TF NF-Y	26	11	8	24	24	7	7	7	4	25	143
CRE	ATF/CREB TF fam.	17	18	8	32	33	32	2	1	0	4	147
E2F	E2F proteins	18	8	4	1	1	7	7	5	2	9	62
ERE	Estrogen receptor	4	20	0	7	6	13	0	7	2	10	69
Ets	Ets TF	41	76	10	28	32	19	49	28	30	20	333
GATA	GATA TF	17	31	8	12	10	13	11	7	1	8	118
LSF	LSF TF	13	19	2	21	20	5	0	4	5	24	113
Mef-2	Myocyte EF 2	57	72	5	43	41	20	7	7	1	21	274
Myc	bHLH and bHLHzip proteins	13	9	4	1	3	11	15	3	0	4	63
Myf	bHLH and bHLHzip proteins	9	24	0	5	4	21	47	38	30	4	182
NF1	NF1 TF	15	29	4	8	8	26	5	5	3	24	127
Sp1	TF in the SP/KLF (kruppel-like factor) fam.	89	61	35	57	53	19	4	13	4	22	357
SRF	Serum response factor	41	44	4	3	6	10	0	1	1	20	130
TATA box	TBP (TATA binding protein), a component of TFIID	75	79	20	25	27	35	7	14	20	34	336
Tef	Transcription EF	7	10	2	18	17	12	0	5	2	5	78

Total		467	535	114	294	293	259	166	149	105	256	2638

ATF: activating transcription factor, CE: cis-elements, CREB: cAMP response element binding, DBRD: DNA binding regulatory domain, DBRP: DNA binding regulatory protein, enhancer factor: EF, family: fam., subfamily: subfam., TF: transcription factor, and TFBS: transcription factor binding Site.
