# Supplementary material for: Comparative Genomics of Herpesviridae Family to Look for Potential Signatures of Human Infecting Strains
Source: Int J Genomics. 2016 May 26;2016:9543274. doi: 10.1155/2016/9543274 (PMC4899598; doi:10.1155/2016/9543274)
Supplement: Supplementary file 1 — Supplementary Figure 1: Partition of the Herpesviridae family pan-genomic matrix into 2 shell, cloud, soft-core, and core compartments. Total gene clusters = 1,785; taxa = 64. Supplementary Table 1: Calculation of cloud, shell, soft core and core genomes. [file 9543274.f1.zip › 9543274.f1.pdf]

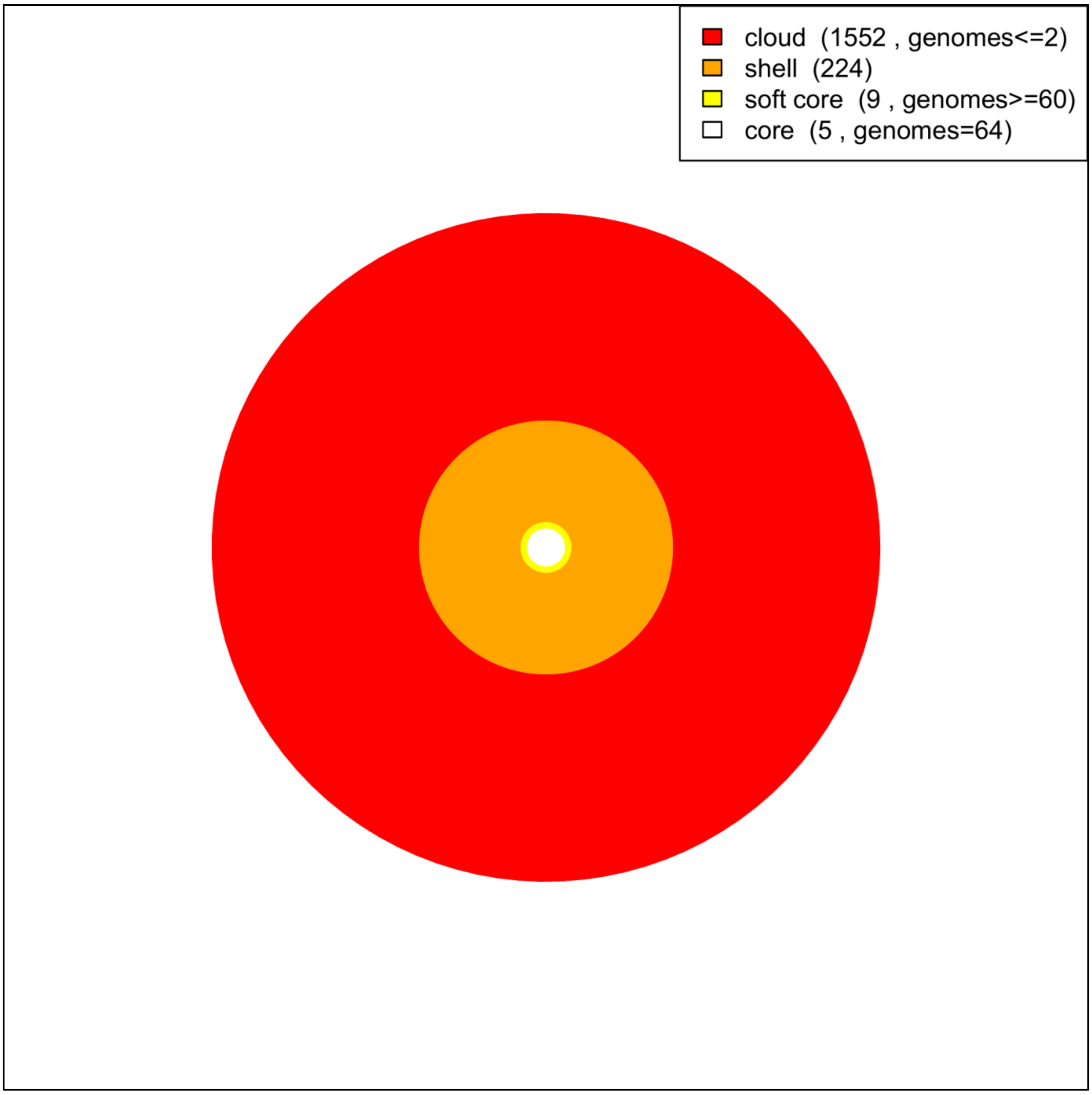

Supplementary Figure 1: Partition of the *Herpesviridae* family pan-genomic matrix into shell, cloud, soft-core, and core compartments. Total gene clusters = 1,785; taxa = 64.

Supplementary Table 1: List of non-human herpes viruses of *Herpesviridae* family used in this study.

**Viral Strain**	**NCBI Accession Number**	**Nucleotide Length (bp)**	**Proteins**	**GC (%)**
***Alphaherpesvirinae* subfamily**				
Duck enteritis virus strain VAC	NC_013036	158,091	77	44.9
Bovine herpesvirus type 1.1	NC_001847	135,301	70	72.4
Bovine herpesvirus 5 strain SV507/99	NC_005261	137,821	70	74.8
Cercopithecine herpesvirus 2	NC_006560	150,715	75	76
Cercopithecine herpesvirus 9	NC_002686	124,784	74	40.5
Chimpanzee alpha-1 herpesvirus strain 105640	NC_023677	153,158	77	68
Equine herpesvirus 1 strain Ab4	NC_001491	150,224	80	56.7
Equid herpesvirus 3 strain AR/2007/C3A	NC_024771	151,601	80	68.1
Equine herpesvirus 4 strain NS80567	NC_001844	145,597	79	50.5
Equid herpesvirus 8 strain wh	NC_017826	149,332	81	54.4
Equid herpesvirus 9 DNA strain: P19	NC_011644	148,371	80	56.1
Falconid herpesvirus 1 strain S-18	NC_024450	204,054	130	61.5
Felid herpesvirus 1 strain C-27	NC_013590	135,797	77	45.8
Fruit bat alphaherpesvirus 1 DNA	NC_024306	149,459	70	60.9
Gallid herpesvirus 1	NC_006623	148,687	79	48.2
Gallid herpesvirus 2 serotype 1 isolate Md5	NC_002229	177,874	85	44.1
Gallid herpesvirus 3 DNA strain: HPRS24	NC_002577	164,270	76	53.6
Cercopithecine herpesvirus 1 strain E2490	NC_004812	156,789	75	74.5
Meleagrid herpesvirus 1 strain FC126	NC_002641	159,160	79	47.6
Cercopithecine herpesvirus 16 strain X313	NC_007653	156,487	75	76.1

Psittacid herpesvirus 1 isolate 97-0001	NC_005264	163,025	77	60.9
Saimiriine herpesvirus 1 strain MV 5-4	NC_014567	156,742	70	67.1
Suid herpesvirus 1	NC_006151	143,461	69	73.6
Testudinid herpesvirus 3	NC_027916	142,494	94	45.9
***Betaherpesvirinae* subfamily**				
Aotine herpesvirus 1 strain S34E	NC_016447	219,474	146	56.3
Caviid herpesvirus 2 strain 21222	NC_020231	233,501	110	55
Cercopithecine herpesvirus 5 strain 2715	NC_012783	226,205	177	51.9
Papio ursinus cytomegalovirus isolate OCOM4-52	NC_027016	226,084	84	51.9
Cynomolgus macaque cytomegalovirus strain Ottawa	NC_016154	218,041	274	49.5
Rhesus cytomegalovirus strain 68-1	NC_006150	221,454	223	49.1
Elephant endotheliotropic herpesvirus 5 strain Vijay	NC_024696	180,800	115	41.5
Elephantid herpesvirus 1 strain Raman	NC_020474	180,421	113	42.3
Mouse cytomegalovirus 1	NC_004065	230,278	161	58.7
Rat cytomegalovirus Maastricht	NC_002512	230,138	167	61
Murid herpesvirus 8 isolate England	NC_019559	202,946	138	46.5
Panine herpesvirus 2 strain Heberling	NC_003521	241,087	169	61.7
Rat cytomegalovirus ALL-03 isolate Malaysian	NC_027200	197,958	123	46.3
Saimiriine herpesvirus 4 strain SqSHV	NC_016448	196,691	141	46.8
Porcine cytomegalovirus strain BJ09	NC_022233	128,367	80	45.5
Tupaia herpesvirus strain 2	NC_002794	195,859	158	66.6
***Gammaherpesvirinae* subfamily**				
Alcelaphine herpesvirus 1 L-DNA	NC_002531	130,608	71	46.2
Alcelaphine herpesvirus 2 isolate topi-AlHV-2	NC_024382	137,090	70	46.6
Ateline herpesvirus 3	NC_001987	108,409	71	36.6
Bovine herpesvirus 4	NC_002665	108,873	79	41.4
Bovine herpesvirus 6 isolate	NC_024303	144,898	79	43.1

Pennsylvania 47				
Callitrichine herpesvirus 3 strain CJ0149	NC_004367	149,696	72	49.3
Rodent herpesvirus Peru	NC_015049	124,335	82	45.2
Equid herpesvirus 2 strain 86/67	NC_001650	184,439	78	57.5
Equid herpesvirus 5 strain 2-141/67	NC_026421	182,380	79	54.7
Cercopithicine herpesvirus 15 strain LCL8664	NC_006146	171,096	80	61.9
Macaca mulatta rhadinovirus 17577	NC_003401	133,719	89	52.5
Murine herpesvirus 68 strain WUMS	NC_001826	119,451	74	47.2
Ovine herpesvirus 2 strain BJ1035	NC_007646	135,135	73	52.9
Saimiriine herpesvirus 2	NC_001350	112,930	76	34.5
