# Supplementary material for: Comparative Genomics of Herpesviridae Family to Look for Potential Signatures of Human Infecting Strains
Source: Int J Genomics. 2016 May 26;2016:9543274. doi: 10.1155/2016/9543274 (PMC4899598; doi:10.1155/2016/9543274)
Supplement: Supplementary file 1 — Supplementary Figure 1: Partition of the Herpesviridae family pan-genomic matrix into 2 shell, cloud, soft-core, and core compartments. Total gene clusters = 1,785; taxa = 64. Supplementary Table 1: Calculation of cloud, shell, soft core and core genomes. [file 9543274.f1.zip › description.docx]

**S1 Materials and Methods**

**S1.1 Materials**

This study focuses primarily on the evolutionary aspects of Herpesviridae family including

HHVs and non-HHVs. Towards this, the strains of non-HHVs included in this study are given in

**Supplementary Table 1**.

**S2 Results**

**Calculation of cloud, shell, soft core and core genomes**

To analyze the genomic evolution of the Herpesviridae family, pan-genome is further

categorized into four compartments including core, soft core, shell, and cloud **(Supplementary**

**Figure 1)**. This compartmentation is based upon the different possible evolutionary mechanisms

as described in the section “Pan-Genome Analysis of Herpesviridae Family” of main manuscript.
